# Effect of a Combination of Low Level Ozone and Metal Ions on Reducing *Escherichia coli* O157:H7 and *Listeria monocytogenes*

**DOI:** 10.3390/molecules18044018

**Published:** 2013-04-04

**Authors:** Suk-Nam Kang, Kui-Jin Kim, Joung-Hyun Park, Ok-Hwan Lee

**Affiliations:** 1 Department of Animal Resources Technology, Gyeongnam National University of Science and Technology, Gyeongnam 660-758, Korea; E-Mail: white@gntech.ac.kr; 2 Department of Cancer and Cell Biology, University of Cincinnati College of Medicine, Cincinnati, OH 45267, USA; E-Mail: kimkj@ucmail.uc.edu; 3 Department of Food Science and Technology, Institute of Food Science, Pukyong National University, Busan 608-737, Korea; E-Mail: pdc327@pknu.ac.kr; 4 Department of Food Science and Biotechnology, Kangwon National University, Chuncheon 200-701, Korea

**Keywords:** ozonated water, metal ion, combination, *E. coli*, *L. monocytogenes*

## Abstract

Ozonated water has been used as a strong antimicrobial agent against foodborne pathogens. In this study, the combined effect of low level ozonated water and different added components, including 0.2% starch and metal ions (1 mM CuCl_2_·2H_2_O and 0.1 mM AgNO_3_), on inactivation of *Escherichia coli* O157:H7 and *Listeria monocytogenes* was investigated. Treatment with 0.4 ppm ozonated water for 30 min resulted in a maximum log reduction in *E. coli* O157:H7 and *L. monocytogenes* compared to initial bacterial counts. The log reductions of bacteria in a starch solution containing ozonated water were slightly higher than those in ozonated water alone. Furthermore, the log reductions of *E. coli* O157:H7 (2.59 and 4.71 log cfu/mL) and *L. monocytogenes* (2.53 and 4.28 log cfu/mL) in a metal ion solution containing 0.2 and 0.4 ppm ozone for 30 min were significantly higher than those of the water and starch added groups (*p* < 0.05). These results indicate that a combination of ozonated water and metal ions may be useful as a antimicrobial agent.

## 1. Introduction

Many methods have been used to preserve and extend the shelf life of food. Physical and chemical methods such as filtration, thermal treatment, and adding chemical agents to extend shelf life have been used to sanitize water and food systems [1]. Microbial contamination usually occurs at the surface of the food gap and linkage part in a food product line; thus, so nontoxic agents should be applied properly to reduce microbes. Chlorine-based agents have often been used to sanitize produce and surfaces of food manufacturing equipment, as well as to reduce microbial populations in water applied for cleaning and packing operations [2,3,4]. Because chlorinated organic compounds such as trihalomethane are potential carcinogens [5], there is a need to investigate the efficiency of non-traditional sanitizers and alternative technologies. Therefore, various preservation technologies, which are healthier for consumers, have been employed [6]. 

Ozone, a strong antimicrobial agent with numerous potential applications in the food industry [7,8,9], has been approved in the US and is classified as a food additive. Ozone has been extensively used to sanitize drinking water for bacteria, molds, viruses, and protozoa [10]. Furthermore, ozonated water has fewer microbes and extends the shelf life of fresh-cut fruits and vegetables [11]. The mode of inactivation by ozone appears to be DNA damage [12,13,14]. Ozone reacts quickly; thus, inactivation occurs by both gaseous ozone (through direct physical contact) and dissolved ozone. A decrease in pathogen numbers, including *Salmonella typhimurium*,* Yersinia enterocolitica*,* Staphylococcus aureus*, *Listeria. monocytogenes*, and *Escherichia coli* O157:H7 has also been described [10,11]. Therefore, the use of ozonated water is an alternative to traditional sanitizers due to its efficacy at low concentrations and short contact times as well as its ability to breakdown toxins to non-toxic products [15,16,17]. Several researchers have reported the effects of organic material and metal ion mixtures on viable microorganisms in water. The objective of this study was to provide basic information on low level ozonation of selected microorganisms including *L. monocytogenes* and *E. coli* O157:H7 and to identify the microbial reduction effect of adding 2% soluble starch and a metal ion solution (1 mM CuCl_2_·2H_2_O, and 0.1 mM AgNO_3_) before ozonation.

## 2. Results and Discussion

### 2.1. Effect of Low Level Ozonated Water on E. coli O157:H7 and L. monocytogenes

Because the ozonation level during food processing should be limited due to the intensive and offensive odor of ozone, we used a low level of ozonated water (<0.4 ppm). Table 1 shows the *E. coli* O157:H7 and *L. monocytogenes* counts in the water at ozone concentrations of 0.1, 0.2, and 0.4 ppm for 10 and 30 min exposures.

The higher the level of ozone used, the greater the log unit reduction in *E. coli* O157:H7 and *L. monocytogenes* counts observed. In the ozonated water containing 0.2 and 0.4 ppm of ozone, the viable microorganism counts of *E. coli* O157:H7 at 10 min (9.29 and 8.40 log cfu/mL, respectively) and 30 min (9.04 and 7.46 log cfu/mL, respectively) were significantly lower than the initial bacterial counts (10.30 log cfu/mL); however, no significant difference was observed in ozonated water containing 0.1 ppm ozone for a 10 min exposure.

**Table 1:** Mean (standard deviation) bacterial counts in log cfu/mL of *E. coli* O157:H7 and* L. monocytogenes* in ozonated water at concentrations of 0.1, 0.2, and 0.4 ppm at 10 and 30 min exposure time.

		Strains			
		*Escherichia coli* ** O157:H7**		*Listeria. monocytogenes*	
	Time	10 min	30 min	10 min	30 min
Dosage					
0 ppm		10.30 ± 0.97 ^a^	10.30 ± 1.14 ^a^	9.67 ± 0.64 ^a^	9.67 ± 0.64 ^a^
0.1 ppm		10.44 ± 1.37 ^a^	9.63 ± 0.61 ^ab^	7.90 ± 0.63 ^a^	8.84 ± 0.36 ^a^
0.2 ppm		9.29 ± 0.20 ^ab^	9.04 ± 0.40 ^b^	6.27 ± 0.19 ^b^	7.65 ± 0.67 ^b^
0.4 ppm		8.40 ± 0.16 ^Ab^	7.46 ± 0.61 ^Bc^	5.27 ± 0.62 ^c^	5.83 ± 0.35 ^c^
Pr > F		*	**	**	**

^a–c^ Values followed by the same letter are not statistically different within the same exposure time in the same microorganism (* *p* < 0.05, ** *p* < 0.01); ^A–B^ Means followed by the same letter are not statistically different within different ozone exposure times in the same microorganism (*p* < 0.05).

In the ozonated water containing 0.2 and 0.4 ppm ozone, the viable microorganisms counts of *L. monocytogenes* at 10 min (7.98 and 6.55 log cfu/mL, respectively) and 30 min (7.65 and 5.83 log cfu/mL, respectively) were significantly lower than the initial bacterial counts (9.47 log cfu/mL). 

The bactericidal effects of ozone have been studied and documented in a wide variety of organisms, including Gram-positive and Gram-negative bacteria as well as spores and vegetative cells [10,14]. The bacterial cell surface has been suggested as the primary ozonation target. Two major mechanisms have been identified during ozone destruction of target organisms [18]. The first mechanism is that ozone oxidizes sulfhydryl groups and amino acids of enzymes, peptides, and proteins to shorter peptides. The second mechanism is that ozone oxidizes polyunsaturated fatty acids to acid peroxides. Ozone degradation of the unsaturated lipid cell envelope results in cell disruption and subsequent leakage of cellular contents. The double bonds of unsaturated lipids are particularly vulnerable to ozone attack. The lipoprotein and lipopolysaccharide layers in Gram-negative bacteria are the first sites of destruction resulting in increased cell permeability and lysis [19]. 

### 2.2. Effect of a Combination of Low Level Ozonated Water and Starch Solution

Many agricultural and food industrial wastes contain starch and cellulose, which are rich in carbohydrates. The complex nature of these wastes may adversely affect biodegradability. Starch-containing solid wastes are easier to process as carbohydrate and hydrogen gas. Starch can be hydrolyzed to glucose and maltose by acid and enzymatic hydrolysis followed by conversion of the carbohydrates into organic acids and then to hydrogen gas [20].

Table 2 shows the *E. coli* O157:H7 and *L. monocytogenes* counts in the 0.2% starch solution containing of 0.1, 0.2, and 0.4 ppm ozone for 10 and 30 min exposures. The higher the ozone concentration, the higher the log reduction of *E. coli* O157:H7 and *L. monocytogenes*. Similar statistically significant *E. coli* O157:H7 populations were observed at 10 min (8.82 log cfu/mL) and 30 min (7.69 log cfu/mL) in starch containing 0.4 ppm ozone.

**Table 2:** Mean (standard deviation) bacterial counts in log cfu/mL of* E. coli* O157:H7 and* L. monocytogenes* in starch solution containing 0.1, 0.2, and 0.4 ppm of ozone at 10 and 30 min exposure time.

		Strains			
		*Escherichia coli* ** O157:H7**		*Listeria. monocytogenes*	
	Time	10 min	30 min	10 min	30 min
Dosage					
0 ppm		10.32 ± 0.97 ^a^	10.32 ± 0.97 ^a^	9.68 ± 0.65 ^a^	9.68 ± 0.65 ^a^
0.1 ppm		10.19 ± 0.94 ^a^	9.87 ± 0.80 ^a^	9.50 ± 0.60 ^a^	9.25 ± 0.50 ^a^
0.2 ppm		9.49 ± 0.33 ^b^	9.21 ± 0.26 ^a^	8.45 ± 0.27 ^b^	7.86 ± 0.54 ^b^
0.4 ppm		8.82 ± 0.13 ^Bb^	7.69 ± 0.54 ^Ab^	7.52 ± 0.43 ^Bc^	6.23 ± 0.49 ^Ac^

^a–c^ Values followed by the same letter are not statistically different within the same exposure time in the same microorganism (*p* < 0.05); ^A–B ^Means followed by the same letter are not statistically different within different ozone exposure times in the same microorganism (*p* < 0.05).

A significant difference in the bacterial counts was observed at 10 min (8.45 and 7.52 log cfu/mL, respectively) and 30 min (7.86 and 6.23 log cfu/mL, respectively) for* L. monocytogenes* in starch containing 0.2 and 0.4 ppm ozone. Interference by organic matter was considered the main reason for the results obtained in this first set of experiments. Sensitivity of microorganisms to ozone is affected by several factors, including the presence of organic matter [21,22]. A high and persistent level of organic substances will have a negative impact on the ozone disinfection rate. 

*E. coli* O157:H7 and *L. monocytogenes* counts in the starch solution group were slightly higher than those in the 0.2 and 0.4 ppm ozonated water groups. Although the initial bacterial count in the starch solution group was lower than that of the water group at all exposure times, it was assumed that adding soluble starch might have been the reason for the interference with ozone activity. Guzel-Seydim *et al*. [23] studied the efficacy of ozone to reduce bacteria in food components using sterile class C buffer and soluble starch in sterile distilled, deionized water. As a result, spore populations were reduced by 4.93 log cycles in buffer, and 4.56 in starch at 10 min. Statistically significant log cycle reductions in the *E. coli* populations at 10 min were observed in buffer (6.10), and starch (6.11). Statistically significant log reductions were detected at 10 min in buffer (6.48), and starch (6.47) for *Staphylococcus* *aureus*.

### 2.3. Effect of a Combination of Low Level Ozonated Water and Metal Ions

Many studies have been conducted on the combined use of copper and silver ions to destroy *Legionella *in cold and hot-water systems at hospitals, with most applications being conducted in recirculating hot-water systems [24,25,26,27]. Metal ions are believed to interfere with enzymes involved in cellular respiration and bind DNA at specific sites. Periodic monitoring of metal ion concentrations has been suggested for potable water to ensure that the concentrations are below the EPA maximum contaminant level of 1.3 mg/L for copper and the life-time health advisory level of 0.1 mg/L for silver, respectively. 

Table 3 shows the bacterial counts of *E. coli* O157:H7 and *L. monocytogenes* in a metal ion solution containing of 0.1, 0.2, and 0.4 ppm ozone for 10 and 30 min. The higher the levels of ozone, the higher the log unit reduction of *E. coli* O157:H7 and *L. monocytogenes* occurred. Statistically significant *E. coli* O157:H7 population differences were observed at 10 min (8.26 and 6.63 log cfu/mL) and 30 min (7.64 and 5.53 log cfu/mL) in the metal ion mixture with 1 mM CuCl_2_·2H_2_O and 0.1 mM AgNO_3_ and containing 0.2 and 0.4 ppm ozone. Significant *L. monocytogenes* population differences were observed at 10 min (7.18 and 5.67 log cfu/mL, respectively) and 30 min (6.59 and 4.83 log cfu/mL, respectively) in a solution containing 0.2 and 0.4 ppm of ozone. The microbial counts in the metal ion solution were higher than those in ozonated water for both bacteria at both exposure times. The reductions of *E. coli* and *L. monocytogenes* in the metal ion solution containing 0.2 and 0.4 ppm ozone were greater than those in the water and starch solution. 

**Table 3:** Mean (standard deviation) bacterial counts in log cfu/mL of *E. coli* O157:H7 and* L. monocytogenes* in metal ion mixture solution (1 mM CuCl_2_·2H_2_O and 0.1 mM AgNO_3_) containing 0.1, 0.2, and 0.4 ppm of ozone at 10 and 30 min exposure time.

		Strains			
		*Escherichia coli* ** O157:H7**		*Listeria. monocytogenes*	
	Time	10 min	30 min	10 min	30 min
Dosage					
0 ppm		10.24 ± 0.95 ^a^	10.24 ± 0.95 ^a^	9.12 ± 0.09 ^a^	9.12 ± 0.09 ^a^
0.1 ppm		10.03 ± 1.04 ^a^	9.02 ± 0.07 ^b^	8.94 ± 0.08 ^Aa^	8.02 ± 0.53 ^Bb^
0.2 ppm		8.26 ± 0.07 ^Ab^	7.64 ± 0.48 ^Bc^	7.18 ± 0.35 ^b^	6.59 ± 0.69 ^c^
0.4 ppm		6.63 ± 0.87 ^c^	5.53 ± 0.89 ^d^	5.67 ± 0.67 ^c^	4.83 ± 0.47 ^d^

^a–c^ Values followed by the same letter are not statistically different within the same exposure time in the same microorganism (*p* < 0.05); ^A–B^ Means followed by the same letter are not statistically different within different ozone exposure times in the same microorganism (*p* < 0.05).

Stout *et al*. [26] indicated that the use of metal ions (0.17 mg/L Cu^++^ and 0.04 mg/L Ag^+^ at distal outlets) was more effective than that of a periodic superheat and flush (temperature of 77 °C in hot-water storage tanks with a 20–30 min flush of outlets) to destroy *Legionella* in a hospital recirculating hot-water system. A similar observation was made regarding the superiority of copper and silver ions to thermal treatment in a hot-water system by Mietzner *et al*. [27]. They also found that copper and silver accumulated at the bottom of the hot-water tanks, which led to a successful, long-term (many months) inhibition of *L. pneumophila*. Intermittent use of copper and silver ions is also successful [26]. Kusnetsov *et al*. [28] indicated that 0.003 mg/L Ag^+^ is sufficient to control growth of *Legionella* in circulating warm water but it was difficult to eradicate *Legionella* from taps and showers.

## 3. Experimental

### 3.1. Preparation of Water Components

The efficacy of ozone to reduce bacterial populations in water was evaluated with soluble starch, CuCl_2_·2H_2_O, and AgNO_3_ (Sigma, St. Louis, MO, USA). A 0.2% starch solution was prepared by adding 2 g of each substrate to a total volume of 1,000 mL sterile high performance liquid chromatography grade deionized water (dH_2_O). A metal ion mixture (1 mM CuCl_2_·2H_2_O and 0.1 mM AgNO_3_) was added to the 1,000 mL sterile dH_2_O. The metal ion mixture required gentle heating with stirring to enter solution. The two components were stored overnight at 25 °C until use.

### 3.2. Preparation of Bacterial Inoculation

Bacterial inoculates were prepared from frozen (−80 °C) stock cultures of *E. coli* O157:H7 KTCC 33150 and *L. monocytogenes* ATCC 15313. The cultures were grown in Luria–Bertaini (LB) broth (Difco, Detroit, MI, USA) supplemented with 0.2% yeast extract. All cultures were grown at 37 °C for 24 h with agitation at 100 RPM provided by a rotary shaker (New Brunswick Scientific, New Brunswick, NJ, USA). The 24 h cultures of each bacterium were washed with 0.1 M sterile phosphate buffer, pH 7.0 three times by centrifugation (1,800 *×g*, 10 min, 21 °C), and the cell pellets were resuspended in the same buffer. All microorganisms were stored under refrigeration until just before use. One mL of microorganism suspension was transferred to 9 mL of 0.1% peptone water (Difco). The diluted cultures were used to inoculate samples in the ozonation experiment. Fresh cultures were prepared for each experimental replication. 

### 3.3. Ozonation of Inoculated Microorganisms

The pilot plant consisted of a CTS^TM^ ozone generator and an ozone analyzer (Samjung Tech. Co. Ltd, Seoul, Korea). The ozone generator output was 5 g/h. The CTS^TM^ ozone injector was used at 30 and 60 min after commencing ozonation in sterile dH_2_O. The injector was stored under dark, refrigerated conditions prior to use. Ozone concentrations in the stock solution were 0.1–0.4 mg/L. Ozone residuals were measured by the indigo colorimetric procedure [29]. Twenty mL of ozonated water (0, 0.1, 0.2, and 0.4 ppm) was added to a sample tube for each of the three components used in this experiment (water, soluble starch, and metal ions). The samples were inoculated with 1 mL of diluted microorganism culture and stored in sealed sterile tubes in the same container as the control samples under agitation. Samples were collected from the tubes at 10 and 30 min after commencing ozonation. This procedure was repeated four times for each bacterium. 

### 3.4. Microbiological Sampling

The samples inoculated with *E. coli* O157:H7 and *L. monocytogenes* were enumerated by plating in LB agar and then incubated at 37 °C for 24 h. All samples were plated in duplicate.

### 3.5. Statistical Analysis

The results were statistically analyzed using analysis of variance and Duncan’s multiple range tests. Statistical significance was accepted at *p* < 0.05.

## 4. Conclusions

In our studies, the ozonated water could be used to reduce the microbial load in fruits and vegetables. In the present study, the efficacy of a combination of low level ozonated water and metal ions on inactivation of *E. coli* O157:H7 and *L. monocytogenes* was investigated. The reduction in the microbe levels with a mixture of low level ozonated water and metal ions was significantly higher than that of individual and combined ozonated water and a starch solution. These results indicate that the effectiveness of sanitizers depends on a combination of ozone concentration and additives including starch and metal ions.
